# Prevalence and Spatial Distribution of Chronic Respiratory Diseases in Dar es Salaam, Tanzania: Hydrological and Household Influences

**DOI:** 10.1029/2025GH001764

**Published:** 2026-06-02

**Authors:** Ibrahim Msuya, Francis Levira, Irene Moshi, Jean O'Dwyer, Gerry F. Killeen

**Affiliations:** ^1^ School of Biological, Earth and Environmental Sciences University College Cork Cork Ireland; ^2^ Department of Health Systems, Impact Evaluation and Policy Ifakara Health Institute Dar es Salaam Tanzania; ^3^ Environmental Research Institute University College Cork Cork Ireland

**Keywords:** chronic respiratory diseases (CRDs), hydroepidemiology, household characteristics, respiratory health, cluster sampled cross‐sectional study, public health

## Abstract

Chronic Respiratory Diseases (CRDs) are a major global health burden, causing significant mortality and morbidity. The World Health Organization reports CRDs led to 4.1 million deaths in 2023, affecting 454.6 million people (5.54% globally). While tobacco smoking is the leading risk factor, others include air pollution, occupational hazards, childhood respiratory infections, and genetic predisposition. This study examines hydrology's role in CRD prevalence and spatial distribution, alongside household‐level factors. Using remote sensing, the Topographical Wetness Index (TWI) was calculated at 30 m resolution and linked to CRD prevalence data from 1,533 households across 15 income‐stratified neighborhoods in Dar es Salaam, Tanzania. Generalized linear mixed models, geovalidation, and ground truthing were employed for analysis. Results showed that CRD prevalence was significantly associated with higher TWI scores (OR = 1.18 [1.04, 1.33] per TWI unit increase, *p* = 0.009), with flood‐prone areas (TWI ≥ 15) exhibiting a ninefold greater risk (OR = 8.63 [2.13, 35]). Other independent risk factors included household size (OR = 1.15 [1.05, 1.25] per person, *p* = 0.003) and communal skip bin use (OR = 4.04 [1.04, 15.80], *p* = 0.045). Conversely, air conditioning reduced CRD risk (OR = 0.22 [0.06, 0.77], *p* = 0.017). Notably, neighborhood income was not directly associated with CRD prevalence after adjusting for other factors, indicating poverty acts as an indirect, rather than proximal, determinant. These findings link moist, mold‐prone environments to CRD risk, especially in urbanizing cities. Poor households often settle in flood‐prone areas due to affordability, underscoring hydrology's role in CRD disparities.

## Plain Language Summary

Chronic respiratory diseases (CRDs) cause serious health problems worldwide, leading to millions of deaths every year. While smoking is a major cause, other factors like polluted air and lung infections also play a role. This study explored how wet, flood‐prone land areas affect CRD rates in Dar es Salaam, Tanzania. We combined detailed satellite data on land wetness with health surveys from over 1,500 households in different income areas. The results showed that people living in wetter, flood‐prone places had a much higher risk of CRD up to nine times more. Larger households and shared waste bins also increased risk, while air conditioning lowered it. Income alone did not directly predict disease risk when other factors were considered. This study highlights how living in damp environments contributes to lung disease, especially for poorer communities in cities facing flooding challenges. Understanding these links can help improve health policies and reduce disease risks in vulnerable urban areas.

## Key Points

- Living in wetter, flood‐prone areas greatly increase the risk of chronic respiratory diseases
- Larger households and shared waste disposal increase disease risk; air conditioning reduces it
- Income alone does not directly predict respiratory disease risk when other factors are considered

## 1 Introduction

Chronic Respiratory Diseases (CRDs) constitute a substantial global health challenge, contributing significantly to both mortality and morbidity on a worldwide scale. According to the World Health Organization (WHO), CRDs accounted for 4.1 million deaths in 2023 and affected approximately 454.6 million people, equivalent to 5.54% of the global population (Momtazmanesh et al., 2023), while in Tanzania, they accounted for 2% of total deaths in 2019 (WHO, 2019b). Historically, tobacco smoking has been the most important risk factor for CRDs at the global level, followed by air pollution, occupational hazards, frequent early childhood lower respiratory tract infections, and genetic predisposition (WHO, 2019a). Over recent years, however, it has become apparent that air pollution has become the most pronounced cause of CRD in towns and cities, with both outdoor elements like dust and particle pollution as well as indoor factors, such as asbestos, paint, cleaning agents, mold and radon, all contributing to chronic obstructive pulmonary disease (COPD) and asthma (Hendryx et al., 2019). Furthermore, these factors exhibit heterogeneity across very fine scales, depending on the geographic location and socioeconomic circumstances of individuals, households, or settlements (Fu et al., 2024).

In the context of the hot and humid coastal equatorial climate of Dar es Salaam city in Tanzania, which has predominantly low‐lying terrain susceptible to waterlogging, hydrology may significantly impact indoor air quality by influencing moisture levels within houses and other residential buildings. Excessive indoor moisture, exacerbated by high temperatures and humidity, provides an ideal environment for mold growth (Fanca et al., 2023). This not only raises esthetic concerns in many houses, but also poses substantial health risks (Mendell et al., 2011). Mold proliferation under such conditions fosters the presence of dust mites, cockroaches and bacteria, all of which contribute to poor indoor air quality (Hasager et al., 2021). However, all of these factors may have different impacts on disparate socio‐economic groups, all of whom live complex lives in diverse physical environments distributed across the city in microheterogeneous mosaics (Msuya et al., 2024).

Recent studies have increasingly highlighted the role of hydrological factors, such as rainfall variability, flooding events, surface water inundation and persistent waterlogging, in shaping environmental conditions linked to respiratory health outcomes (Peirce et al., 2022; Xu et al., 2025). Flood‐prone environments have been associated with elevated indoor dampness, mold proliferation and increased exposure to bioaerosols, all of which are recognized contributors to CRD (Groot et al., 2023; Holden et al., 2023). Similarly, inadequate drainage infrastructure and recurrent flooding in rapidly urbanizing environments have been shown to exacerbate household moisture accumulation and environmental exposures relevant to respiratory morbidity (Vélez‐Torres et al., 2022). However, most previous studies have relied on relatively coarse climatic indicators, such as rainfall totals or flood occurrence records, with limited attention to fine‐scale spatial hydrological variability within urban environments. Addressing this gap requires spatially explicit hydrological indicators that can capture fine‐scale environmental wetness patterns and their potential influence on respiratory health outcomes. The Topographic Wetness Index (TWI) provides a spatially explicit proxy for potential surface moisture accumulation and drainage characteristics, and has recently been applied in urban flood susceptibility and hydrological modeling studies (Allende‐Prieto et al., 2024; Gupta et al., 2024).

Building on this perspective, previous studies have linked damp and moldy housing to long term CRDs risk (Burr et al., 1988; Gravesen, 1972; Lumpkins et al., 1973). Similarly, other studies have associated it with the age of the house (de Marco et al., 2013; Li et al., 2020; Mishra et al., 2023) and general air quality (Annesi‐Maesano et al., 2021; Bălă et al., 2021; Monoson et al., 2023) but only a few have as yet identified underlying physical geographical factors driving these hazards. This study investigated the role of hydrology, using the TWI as a spatial proxy, as a potential determinant of the prevalence and spatial distribution of CRD, while also accounting for other household‐level factors commonly reported in the literature.

## 2 Method

### 2.1 Study Site

**Figure 1:**
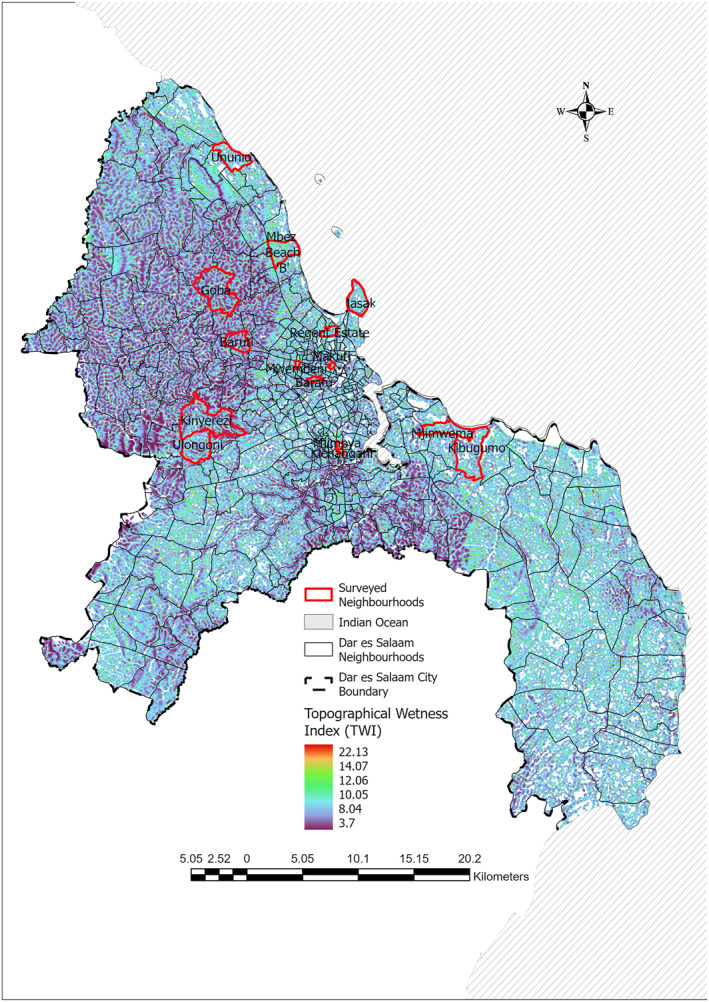
Distribution of sampled neighborhoods (*n* = 15) in the survey conducted in Dar es Salaam city and reported herein (Appendix C), overlayed with a topographical wetness map at a resolution of 30 m.

This cross‐sectional study, a household survey supplemented with a hydrological survey using remote sensing data, was conducted in 15 income‐stratified neighborhoods (mitaa, literally translated as streets) of Dar es Salaam, the largest city in Tanzania. The neighborhoods have a diverse and complex pattern of income and poverty distribution across fine spatial scales (Msuya et al., 2024). Situated on Tanzania's Indian Ocean coast, Dar es Salaam is known for its economic importance and large population (Todd et al., 2019). At the time of the last population and housing census in 2022, Dar es Salaam spanned an area of 1,493 km^2^ with a population of 5.4 million residents, accounting for 8.7% of the country's population (URT, 2022). The city experiences a coastal equatorial climate characterized by consistently high temperatures and humidity. The city primarily occupies low‐lying terrain along a relatively flat, largely sandy, coastal plain intersected by several small rivers, with many areas within these valleys prone to water accumulation and flooding (Figure 1). Data collection took place between June and September 2021.

### 2.2 Participants

Households were used as the primary units of analysis, with neighborhoods serving as the most suitable geographic sampling unit because they are both large and small enough to capture heterogeneity within and between such cluster samples. The study aimed to sample representative clusters of neighborhoods from all across the city, thus maximizing their diversity at this geographic scale rather than deliberately including any particular ethnic groups or population segments. The selected neighborhoods in the study area spanned most of the social and spatial continuum within the city, ranging from affluent to deprived, inner city to urban periphery, and planned to unplanned. Hence, a sample size of at least 1,500 households distributed across five income bands in 15 neighborhoods with 300 households in each neighborhood income category within the city was considered sufficient to represent the social and spatial spectrum dynamics within the neighborhoods regardless of the city population. Also based on the old urban planning assumption that neighborhood population ranges from 5,000 to 7,000 (Baffoe, 2019), a sample size of around 364 individuals was considered sufficient to achieve prevalence estimates with a 5% margin of error for a neighborhood with a population of 7,000 (Charan & Biswas, 2013).

The sampling strategy employed in this study therefore followed a multi‐stage approach, starting by stratifying neighborhoods into income categories ranging from low to high. A participatory stratification approach based on consensus subjective perspectives of local community representatives, town planners and technical experts (Msuya et al., 2024) was used to categorize every neighborhood in Dar es Salaam into five income strata (low, mixed low and medium, medium, mixed medium and high, and high). In the first stage, three neighborhoods were selected from within each income category by population‐weighted random sampling obtained from the census data, comprising 15 widely distributed neighborhoods overall (Figure 1). Following this, dwellings were selected systematically at a predefined interval (*n*). The value of *n* represented the number of dwellings skipped between surveyed dwellings in each neighborhood, which depended on the total number of sampled dwellings and the total dwellings (*n* = total dwelling/total sampled dwellings), ranging from 70 to 105 households. One household, defined as a group of individuals living together and sharing meals, was randomly selected from within each selected dwelling using a household list and the random selection generator on a mobile device. In cases where household members were absent during the initial visit, interviewers revisited the following day, leaving contact information for the interviewer at the door of the household head. Finally, respondents were selected using one of two distinct approaches, either by (a) listing all household members aged 18 years and above and then allowing an ODK tool to randomly select one respondent for the interview, or by (b) applying the birth date rule, whereby the next household member whose birth date was nearest was interviewed. Similar to the previous stage for the household head, interviewers returned on a subsequent day if household members were unavailable during the initial visit. In accordance with the approved protocols, written consent was obtained from all human participants.

### 2.3 Procedures

A total of 17 experienced Tanzanian research assistants (RAs) were recruited and trained for 5 consecutive days from 17th to 21st May 2021 at the Kibaha Conference Centre, Pwani Region, Tanzania. A 2‐day pilot of the survey tools and feedback session was conducted to improve the survey. The RAs were then divided into small groups of 3 people, with a supervisor for each group to oversee all research fieldwork activities. A structured questionnaire, consisting of various sections, was employed. These sections covered details such as the household roster (including information on sex, age, education, marital status, relationship to the household head, employment status, and long‐term illness), as well as aspects related to household characteristics, health, education, income, neighborhood, and gender. During the household survey, respondents were specifically asked whether any household member had been diagnosed with any chronic respiratory disease. These included but were not limited to, asthma, COPD and interstitial lung diseases. Additionally, respondents were queried about other non‐communicable diseases, such as cancer, diabetes, heart diseases, kidney diseases, high blood pressure, back pain, mental health conditions, and any other sundry relevant diagnoses.

### 2.4 Hydrology Data Acquisition

A 30 m resolution Digital Elevation Model (DEM) of Dar es Salaam was adopted to generate the Topographical Wetness Index (TWI) using the ArcGIS Pro 3.2.2 hydrology spatial analyst tool. The DEM underwent several preprocessing steps to ensure its suitability for hydrological analysis. These steps included artifact correction and resampling. Subsequently, slope values were computed for each pixel in the DEM using the arctan function, which converted elevation differences between neighboring pixels into radians (Hojati & Mokarram, 2016). These radians were later transformed into degrees for ease of interpretation. Following the slope calculation, the contributing upslope area for each pixel was determined using flow accumulation algorithms, primarily implemented through D∞ flow accumulation methods. This process involved calculating the upstream contributing area per unit width orthogonal to the flow direction. Finally, the TWI was computed using a logarithmic formula that integrates the slope and the contributing upslope area per unit width. Specifically, this entailed calculating the natural logarithm of the ratio between the upslope contributing area and the tangent of the slope angle. The generated TWI values provided information about each pixel's relative wetness or water accumulation potential. The raster calculator tool was used to compute TWI values for each 30 × 30 m pixel by incorporating the derived slope and upslope contributing area, obtained from the DEM and flow accumulation data, respectively (Ali & Abdulateef, 2013).

### 2.5 Data Analysis

**Figure 2:**
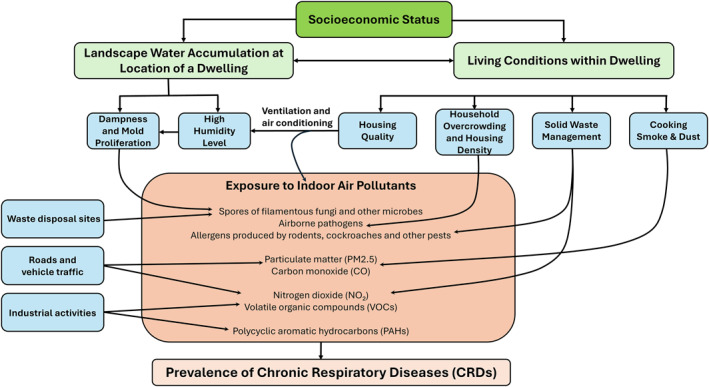
A hypothetical causal pathway diagram illustrating the underlying conceptual framework used to inform the interpretation of this analysis, based on careful literature review and consideration of the processes that are understood to influence chronic respiratory disease risk (Falkowska, 2016; Hendryx et al., 2019; Lim et al., 2021; Munyai & Nunu, 2020).

TWI values for each surveyed household were calculated for each of 1,533 spatially referenced household coordinates using the extract values to points tool to extract raster values for specific point features in the ArcGIS Pro 3.2.2 analyst tool, with the lowest estimated TWI being 5.4 and the highest being 20.1 (Appendix D), for driest and wettest households, respectively. Although it was hypothesized a priori that TWI was an important underlying distal cause of CRD (Figure 2), the following regression analyses were conducted with systematic and unbiased forward stepwise selection procedures to determine whether it was independently associated with disease prevalence even when other factors known to influence risk (Figure 2) were objectively accounted for.

Using R 4.3.3 and RStudio 2023.12 for statistical analysis, binary CRD prevalence outcome data for individual households were related to the TWI estimates for that location together with other recorded factors in the household survey using general linear mixed models. Geographic variation associated with unidentified neighborhood‐specific characteristics was accounted for by including neighborhood as a random effect. Univariate and then multivariate analyses were conducted to explore the association between CRD prevalence and the studied independent variables, with the best fit multivariate model identified using a systematic forward stepwise selection procedure. The building of the best‐fit multivariate model started with univariate analysis, where variables were sequentially added to the model in order of their initial *p* values, starting from the lowest and proceeding through to the highest. Beginning with an empty model with no predictors, each potential predictor variable provisionally added to the model was evaluated based on its contribution to improving the model fit, using statistical criteria such as the Akaike Information Criterion (AIC) the Bayesian Information Criterion, or *p*‐values whereby the predictor that provided the most significant improvement in the model's fit was retained in the model. This process was repeated iteratively, with the model being re‐evaluated after each new predictor was included until no remaining variables offered a statistically significant improvement to the model based on the aforementioned criteria. This forward selection method ensured that the final model included only those predictors that significantly contributed to explaining the variability in the response variable, thus striking a parsimonious balance between model complexity and explanatory power. Note that even variables that did not approach significance in the univariate analysis were each tentatively added in order of their univariate *p* values to the multivariate model to explore whether their inclusion could add incremental explanatory power to already robust multivariate models that allow for far more other sources of variance than the original univariate models, thereby uncovering nuanced relationships that would not otherwise be obvious.

While contemporary best statistical practice often constrains such model building processes based on the a priori hypothesis or hypotheses that motivated the study (Bojinov & Dominici, 2024; Lederer et al., 2019; McNamee, 2005; Westreich & Greenland, 2013), taking that approach made absolutely no difference to the stepwise model selection process in practice. This occurred because TWI was also identified as the strongest predictor of CRD risk, with the lowest AIC and *P* values, in the first round of simple univariate analyses of each individual potential explanatory variable, so it was the first independent variable to be included in the multivariate model at the very outset of the forward stepwise selection procedure applied.

**Figure 3:**
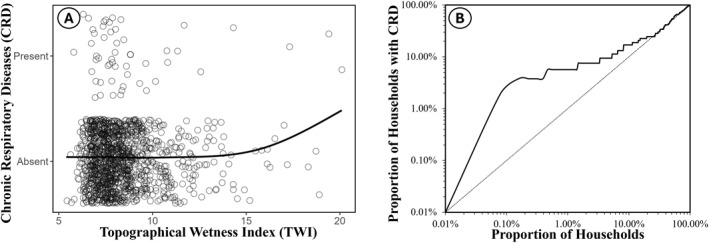
Graphical illustration of chronic respiratory diseases (CRD) risk concentration in a relatively small fraction of houses with unusually high topographical wetness index (TWI). Panel (a) Jitter plot and smoothed moving average curve (LOESS regression) showing the nonlinear relationship between chronic respiratory disease (CRDs) and topographical wetness index (TWI). Panel (b), Log‐Scaled area under the curve plot (AUC) plot narrative interpretation showing the cumulative proportion of households with one or more CRD cases and proportion of total households with both ranked from highest to lowest TWI values.

To identify the optimal approach for modeling the variables to maximize statistical power, several different transformations or reclassifications of these variables were included and compared in distinct univariate models to determine which format of each variable yielded the best fit. Several TWI transformations were assessed as potential predictors of CRDs, specifically the logarithm, square root and binary classification as either above or below 9 (See Results for justification of this cutoff). After interpreting this a priori analysis and examining the smoothed curve relationship between CRD prevalence and TWI (Figure 3), a further categorical analysis of the influence of this variable was conducted post hoc by stratifying into households at locations where TWI ≥ 15 and those where TWI < 15. The number of people in the household was also assessed as an ordinal categorical variable by classifying it into four groups: <5, 5 to 9, 10 to 14, and ≥15 occupants. Moreover, both energy sources were combined and assessed as a single binary variable to represent households using either charcoal or canned gas as relatively clean fuels for cooking. In addition, the neighborhood income variable was also assessed in a reclassified form, reducing based on their frequency distribution from five categories (low, mixed low and medium, medium, mixed medium and high and high) to three (low, medium and high) by combining low with mixed low and medium as a single low category, and mixed medium and high with high as a single high category.

To validate the model results, high‐resolution satellite images from May 2021, taken near the time of data collection, were used to georeference the hydrological characteristics of areas where CRD cases were identified. These findings were further corroborated by ground truthing in the same locations. Whereby the findings from the study were verified by physically visiting the same locations where the data had been collected and cases of CRDs identified. This on‐site verification compared and confirmed the accuracy of the remote (TWI) and modeled data findings against actual household and neighborhood hydrological conditions. The validated examples of local visual ground truthing presented herein represent a subset of many assessed during the analysis, which were selected to reflect the broader cartographic and hydrological variability across the city, rather than selectively chosen cases. All findings were considered, including unexpected results, which were reported and further investigated through ground‐truthing. In fact, such detailed qualitative examination of locations that deviated from predictions of the quantitative analysis that turned out to be the exceptions that intuitively proved the rule.

### 2.6 Ethical Considerations

This study was conducted in accordance with the ethical principles of the Declaration of Helsinki. An ethical clearance for the study, a component of the Sustainable Cities project at the Ifakara Health Institute (IHI), was granted by the Institutional Review Board of the Ifakara Health Institute (IHI/IRB/NO: 26‐2018) and the Medical Research Coordination Committee of the National Institute for Medical Research in Tanzania (NIMR/HQ/R.8a/Vol. IX/2954).

Written consent, in accordance with the approved protocols, was obtained from all human participants with detailed information about the survey's purpose, procedures, potential risks, and benefits, allowing them to make an informed decision about their involvement. Confidentiality was maintained by anonymizing data and securely storing personal information to prevent unauthorized access. To minimize harm, the survey was designed to avoid sensitive topics in the context of the study that could cause distress, and participants were given the option to skip questions or withdraw at any time without penalty. Transparency was upheld by clearly communicating the survey's objectives, methods, and the use of collected data, ensuring participants understood how their information would be used and the overall aim of the research.

### 2.7 Role of the Funding Source

Data used in this study were collected from the research conducted as part of the Center for Sustainable, Healthy, and Learning Cities and Neighborhoods, supported by funding from the United Kingdom Research and Innovation (UKRI) through the Economic and Social Research Council (ESRC) under the UK Government's Global Challenges Research Fund (GCRF), through a project entitled Sustainable, Healthy, and Learning Cities and Neighborhoods. The funder had no role in the designing of the study or the collection, analysis, and interpretation of data.

## 3 Results

A total of 1,533 households with approximately 7,098 residents were interviewed, representing a mean household size of 4.6 members. Out of 1,468 surveyed households that responded to the subsection on non‐communicable diseases, 65 reported one or more diagnosed cases of CRD (Appendix B), making a total of 4.4% prevalence. The age and sex distribution of the study population are presented in Appendix A and a detailed descriptive analysis table showing number of outcomes events of each variable investigated in the current study stratified into income categories used in sampling the study neighborhoods are found in Appendix B. While six of the nine factors investigated were associated with CRD in the univariate analysis, only four were included in the final best‐fit multivariate model (Table 1). In all these univariate and multivariate models, unidentified neighborhood‐specific characteristics accounted for considerable variation that was captured by a corresponding random effect.

**Table 1:** The Results of Univariate and Multivariate Logistic Regression Analyses (GLMM) Investigating Factors Apparently Associated With the Prevalence of Chronic Respiratory Diseases (CRDs) in Dar es Salaam City, Tanzania

Fixed effects	Statistical parameter					
	Univariate			Multivariate		
	OR [95% CI]	*t*‐value	*P*	OR [95% CI]	*t*‐value	*p*
Topography and hydrology						
Topographical Wetness Index (TWI)^a^	**1.16 [1.09, 1.24]**	**4.380**	**<<0.00001**	**1.18 [1.04, 1.33]**	**2.603**	**0.00925**
Household Characteristics						
Number of people in the household^b^	**1.14 [1.04, 1.24]**	**2.946**	**0.00322**	**1.15 [1.05, 1.25]**	**2.934**	**0.00335**
Recent birth in the household	**1.82 [1.08, 3.08]**	**2.233**	**0.0255**	1.42 [0.76, 2.65]	1.097	0.273
Any air conditioning	**0.40 [0.18, 0.86]**	**−2.328**	**0.0199**	**0.22 [0.06, 0.77]**	**−2.379**	**0.0174**
Use of a communal skip	**3.86 [1.01, 14.74]**	**1.972**	**0.0486**	**4.04 [1.04, 15.80]**	**2.006**	**0.0449**
Years lived in a dwelling	1.00 [1.00, 1.01]	0.911	0.362	1.00 [1.00, 1.01]	1.828	0.0676
Cooking Energy^c^						
Use of charcoal	**1.93 [1.08, 3.44]**	**2.218**	**0.0266**	1.35 [0.69, 2.62]	0.876	0.381
Use of canned gas	0.61 [0.34, 1.09]	−1.669	0.0951	0.80 [0.40, 1.59]	−0.641	0.522
Neighborhood Income^d^	0.84 [0.52, 1.36]	−0.702	0.483	0.84 [0.45, 1.56]	−0.551	0.581
Random Effect				σ	SD	
Street Name				1.073	1.036	

*Note.* OR: Odds Ratio. 95% CI: 95% Confidence Interval. *p*: Estimated probability of the null hypothesis being true. *σ*: Variance. SD: Standard Deviation. The bolded values indicate variables that are statistically significant (*p* < 0.05), meaning they have a reliable and independent association with the outcome and are unlikely to be due to random chance.

^a^ In addition to the simple logit‐linear relationship reported here, a number of TWI transformations were assessed as potential predictors of CRDs, specifically the base 10 logarithm, square root, and binary classification as either above or below 15. However, all these alternative formats yielded models with poorer goodness of fit (AIC ≥ 409).

^b^ The number of people in the household was also assessed as an ordinal categorical variable by classifying it into four groups: ≤4, 5 to 9, 10 to 14, and >15. However, this yielded a model with poorer goodness of fit (AIC ≥ 413).

^c^ Both energy sources were combined and assessed as one variable to represent households using either charcoal or canned gas for cooking in a binary form. However, no model convergence was attained.

^d^ The neighborhood income variable was also assessed following reclassification to yield 3 categories instead of 5, with mixed low and medium income being combined with low, and mixed medium and high income being combined with high, while the medium was retained as it was, making three categories called low, medium, and high. However, this also did not yield a model with improved goodness of fit either (AIC ≥ 409).

In addition to the simple logit‐linear relationship reported in Table 1, a number of TWI transformations were also assessed as potential predictors of CRDs. However, all these alternative formats yielded models with poorer goodness of fit (AIC ≥ 409) than that described in Table 1 for the final best‐fit model (AIC = 407). The number of people in the household was also assessed as an ordinal categorical variable but also yielded a model with poorer goodness of fit (AIC ≥ 413). Similarly, attempts to recode energy sources yielded a model that failed to converge and reclassification of neighborhood income into only three categories from five, also resulted in poorer goodness of fit (AIC = 409). Hence, all results presented herein are based on variables analyzed in their original format and scale because this maximized statistical power.

Reassuringly, even though TWI was assumed beforehand to be a distal case of CRD and every effort was made to keep the multivariate model selection process systematic, objective and unbiases, it exhibited an apparently robust influence upon CRD, being consistently the first explanatory variable to be included and most clearly justified for retention The final multivariate analysis indicated a clear positive association between hydrological determinants of wetness and household CRD prevalence, whereby the risk of CRD increased by 18% per TWI unit increment (Table 1). Further categorical analysis of TWI after post hoc stratification was therefore informed by Figure 3, wherein a steep response curve was seen only in the very wettest locations (TWI ≥ 15). In these particularly flood‐prone locations (≥99th percentile), CRD risk was approximately 9‐fold greater than elsewhere (OR [95% CI] = 8.63 [2.13, 35]) when the influence of household size, air conditioning and communal waste disposal were all accounted for. Also, the number of people living in the household and CRD prevalence indicated a positive association, with risk increasing by about 15% per extra household member. In addition, the use of communal skip bins in the household was associated with four‐fold greater CRD, indicating an important influence on outdoor air quality. On the other hand, however, the use of air conditioning was associated with CRD being reduced approximately four‐fold.

While having recently had a birth in the household and the use of charcoal both appeared positively associated with CRD risk in the univariate analysis, neither was included in the final multivariate model. Interestingly, the number of years a household has lived in the dwelling emerged as a CRD risk factor as the addition of other independent variables improved the statistical power of the multivariate model. While univariate analysis indicated no association, the final multivariate model indicated an association that approached significance. Nevertheless, it did not quite reach statistical significance or improve the model AIC and was therefore excluded from the final model (Table 1). The use of canned gas was not found to have any association in either univariate or multivariate analysis. Interestingly, neighborhood income was not associated with CRD prevalence when the effect of all these other risk factors was accounted for, confirming that poverty is an indirect, distal cause of CRDs rather than a direct, proximal one.

The results in Table 1 demonstrating the clear association of CRD with hydrological likelihood of local water accumulation is further illustrated by Figure 3a, which shows an uneven distribution of TWI values across a wide range, with substantial clustering at the lower end, between 6 and 12. The fitted moving average indicates a generally flat trend for low to moderate values of the TWI, suggesting consistently low CRD prevalence within this range. However, a noticeable upward trend in this curve at TWI values above approximately 15 suggests an increase in disease prevalence at higher levels. This consistent non‐linear relationship was identified assuming any specific model form strongly implying there is an authentic threshold effect and steady upward trend in risk with increasing TWI. Panel b, log‐scaled area under the curve (AUC) graph reinforces this interpretation, with cumulative proportion of households with CRD and the proportion of total households ranked from highest to lowest TWI, exhibiting a steep slope at the beginning of the curve, indicating a high concentration of CRD in a small proportion of households.

**Figure 4:**
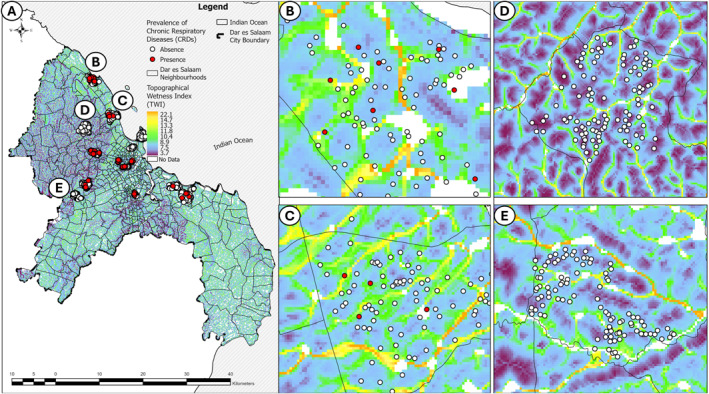
Spatial distribution of chronic respiratory diseases (CRDs) in the surveyed neighborhood's clusters of households in Dar es Salaam city, (a) with high resolution view of representative areas with either high (b, c) or low (d, e) CRD prevalence and TWI. The map presents a topographical wetness index (TWI) overlay to show relationships of potential hydrological factors influencing CRD prevalence. Panels (b) and (c) shows neighborhoods with generally elevated TWI where households with reported CRD diagnoses that were usually found in areas with or very near to those with high estimated TWI. Panels (d and e) illustrate neighborhoods without reported CRD diagnoses in areas with predominantly low TWI.

These results are further supported by cartographic representations of CRD distribution across several areas of Dar es Salaam city, as depicted in Figure 4. Many households with reported cases are found in or beside areas with relatively high TWI (panels b and c), while areas without diagnosed cases are predominated by relatively low TWI (Panels d and e). Nevertheless, it is also obvious from Figure 4 that some CRD cases are found in areas with relatively low estimates, so further visual investigation of potential risk factors beyond TWI estimates was undertaken.

**Figure 5:**
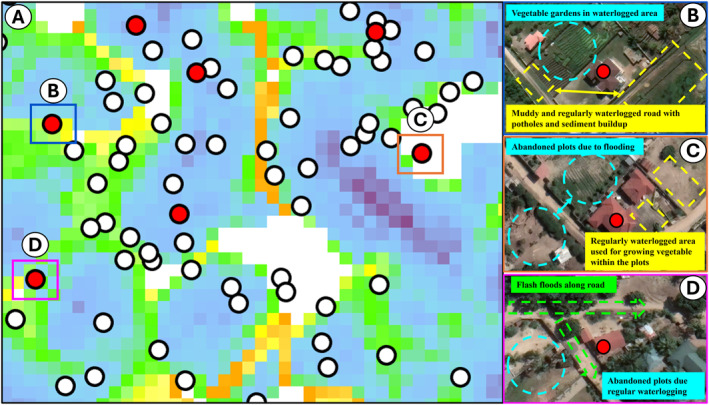
Visual cartographic geovalidation of distribution of chronic respiratory diseases (CRD) over the topographical wetness index (TWI). Panel (a) shows how three cases of CRD are obviously located in areas of high TWI, while panels (b–d) geovalidated this interpretation with high‐resolution satellite image showing fine scale landscape features consistent with regular waterlogging and flash floods.

Careful examination of high‐resolution satellite images, taken close to the time when data were collected (May 2021) to ensure the compatibility, showing the obvious spatial association of CRD cases with visual evidence of regular waterlogging in areas of elevated TWI (Figure 5, panel a).

**Figure 6:**
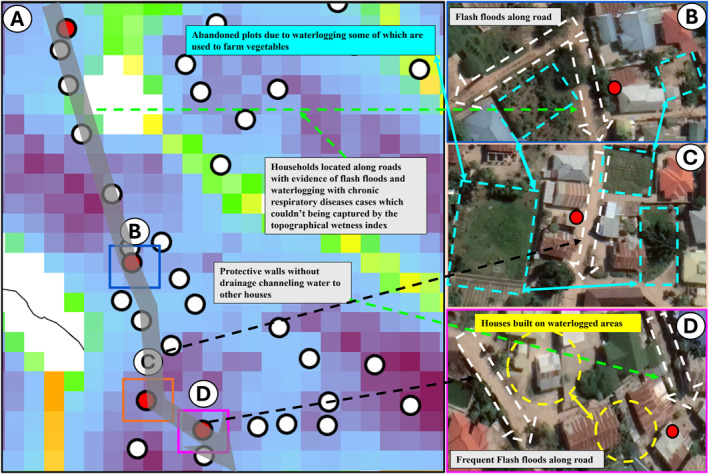
Further geovalidation that distribution of chronic respiratory diseases (CRD) prevalence is strongly influenced by hydrological phenomena even where they occur at residual scales too fine to be detected by topographical wetness index (TWI). Panel (a) shows a series of cases of CRD located in a low TWI that were noted to occur along a rarely straight transect, which proved to be a road when the matching high‐resolution image was examined. While panels (b–d) shows an overlay with high resolution satellite image geovalidating fine scale hydrological features which were not be captured by TWI showing effect of fine scale hydrological processes that clearly influence CRD.

Furthermore, the results in Figure 6 showed that coarse scale TWI initially failed to capture the hydrological nature of some households (a), as some with CRD were found in areas with low estimated TWI. However, a clear effect was observed when they were geovalidated with fine scale landscape features (b–d).

**Figure 7:**
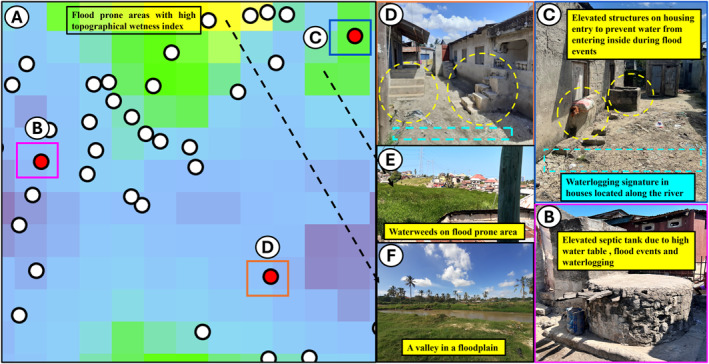
Geovalidation and ground truthing (field verification) of hydrological conditions in different housing blocks with evidence of diverse housing typologies, construction methods and water accumulation in areas with reported diagnosed cases of chronic respiratory diseases (CRD). Panel (a) show a block which is relatively wet, flood prone area with high TWI with its typical housing typologies and surrounding environment in panels (b–f). Panels (d and c) presents construction method used to protect water from entering the houses during flood but also showing evidence of water accumulation on the ground. In addition to that even septic tanks and soak aways pits have been elevated due to high water table and numerous flood incidents (panel b). But also, some of the houses with CRD are found within the catchment areas of the valleys (60 m), as it can be seen in panel a with evidence of water weeds and flooding valley in panels (e and f).

These obvious instances of clear geographic association between CRD cases and visual indicators of water accumulation were further been reinforced by using both geovalidation and ground truthing (field verification), as illustrated in Figure 7.

Hence, all these results confirmed by geovalidation and ground truthing with reference to the model results in Table 1, indicated that the presence of CRD is not merely a random occurrence rather than presentation of evident spatial pattern closely associated with hydrology.

## 4 Discussion

This study demonstrated the role of hydrology as a clearly important fine‐scale determinant of CRD prevalence while also accounting for other household‐level factors related to air quality in indoor and outdoor environments. The association of CRD prevalence with hydrological conditions, as estimated based on water accumulation likelihood (TWI), apparently confirms it to be an important underlying cause of persistent dampness and moisture in housing structures built in low‐lying terrain (Figure 4). Such conditions clearly not only affect the building's aesthetics, but also indoor air quality and respiratory health. A wide range of bacteria and fungi, including filamentous fungi, often referred to as mold, proliferate inside buildings that are continuously moist. These microbes discharge toxic volatile organic compounds (VOCs), spores, and broken cells into the atmosphere, all of which are harmful to human health, and respiratory health in particular (Hernberg et al., 2014; Heseltine & Rosen, 2009). These results corroborate the findings of a great deal of the previous work on the risks of dynamic temporal excessive rainfall and flooding to respiratory health, (Mulder et al., 2019; Paterson et al., 2018; The Lancet Respiratory, 2024) by bridging the gap in understanding the topographic features which exacerbate the static spatial risk of CRD.

The findings of this study also support existing knowledge relating to the influence of overcrowding on indoor air quality and CRD risk. For example, several studies have shown that there is a connection between indoor air quality and household size. (Gilbey et al., 2023) Although there may not be a direct causal relationship between the number of individuals living in a household and CRD, cooking habits, fuel use, and exposure to indoor pollution, all likely contribute to this relationship as the actual proximal causes, with the likelihood of negative health consequences from indoor air pollution increasing with household size. (Kovesi et al., 2007; Lim et al., 2021)

On the other hand, one risk factor identified in this study relates to outdoor air quality, specifically the use of communal skips for disposing of household waste. Similar to other reports, our findings indicated a notable association between the use of a communal skip and CRD prevalence, implying direct impacts of waste disposal practices upon respiratory health (Boadi & Kuitunen, 2005). Communal skips serve as prominent collection points within neighborhoods, allowing households to dispose of various types of waste. However, the accumulation of waste within these bins can significantly affect air quality (Boadi & Kuitunen, 2005; Gao et al., 2023). The proximity of communal skips to residential buildings may contribute to outdoor air pollution by releasing VOCs, particulate matter, and other pollutants during the decomposition of waste materials (Munyai & Nunu, 2020). While it may appear intuitive to attribute outdoor air pollution solely to industrial sources or vehicular emissions, this study underscores the role of waste disposal practices in elevating pollution levels, with the risk of CRD in households using communal skip bins being four‐fold higher. This could have been caused by the malfunctioning municipal services for collecting waste from communal skip bins, leading to the accumulation and overflow of refuse over time. (Kassim & Kayaga, 2016; Mhache, 2012) VOCs emit toxic pollutants like ammonia, methane, and hydrogen sulphides, that compromise air quality and intensify respiratory problems in residential neighborhoods. (Domingo et al., 2020; Falkowska, 2016; Ferronato & Torretta, 2019)

The exhaustive, objective forward stepwise selection process applied revealed that, when all the other significant predictors of prevalence were accounted for, household duration of residence in the same dwelling approached significance as a risk factor for CRD (Table 1). While subtly different in terms of the question asked, this finding is nevertheless broadly consistent with existing evidence that CRD risk increases as buildings age and deteriorate. Unless rigorously maintained, aging buildings become more susceptible to mold growth, and poor air quality more generally, due to factors like deteriorating insulation, increased moisture penetration and inadequate ventilation (Chaudhari et al., 2023).

However, one of the findings presented in Table 1 challenges some established perspectives in the field of respiratory health. While using air conditioning is generally thought to exacerbate respiratory illness, Table 1 indicates that it actually reduces CRD risk more than four‐fold in Dar es Salaam. Fortunately, these surprising findings may be rationalized by understanding the physical effects of air conditioning on the indoor atmosphere in such a coastal equatorial climate. While ambient relative humidity (RH) levels in Dar es Salaam and many other topical cities routinely exceed 60% saturation, providing a favorable environment for the formation of mold (Bomberg & Brown, 1993; Coppock & Cookson, 1951), air conditioning removes moisture from the air through cooling and consequent condensation. Maintaining indoor RH levels within the recommended range of 40%–60% not only keeps the occupants immediately comfortable, but it also prevents mold proliferation within that dwelling (Alaidroos & Mosly, 2023; Jones et al., 2022; Wolkoff, 2018; Yu et al., 2009). Furthermore, modern air conditioning units equipped with specially designed dry mode features deliberately dehumidify the indoor environment by operating at lower fan and compressor speeds (Afram & Janabi‐Sharifi, 2014). Having said that, various details of how air conditioning systems operate may result in negative impacts on respiratory health, such as short cycling, refrigerant leaks, dirty air filters, poor maintenance, dysfunctional humidity control, excessive cooling, improper sizing and the presence of allergens or existing mold growth, as well as pre‐existing health conditions, are important to consider before simplistically recommending it as a panacea for respiratory health in the tropics (Chirico et al., 2020; Lenzer et al., 2020).

On the other hand, while several other variables initially appeared to be significant in univariate analysis, they did not even approach significance when other, more robust, predictors of CRD prevalence were accounted for by more powerful and nuanced multivariate models (Table 1). Also, having had a recent birth in the household was not identified as a risk factor for CRD when the effects of other clear predictors were accounted for by the multivariate analysis presented herein (Table 1), While this might initially appear to contradict established evidence for higher risk of CRD among newborns, (Buchwald et al., 2019; Marangu & Zar, 2019) this may be readily explained by intuitively obvious covariance between the presence of newborns and the overall number of people in the household, presumably resulting in the latter assimilating variance associated with the former in the model presented in Table 1.

Furthermore, despite many studies describing the role of the source of cooking energy as a contributor to air pollution in households that is clearly associated with CRD (Chan et al., 2019; Jindal et al., 2020; Langbein, 2017; Torres‐Duque et al., 2008), no such association was observed here. While charcoal use was associated with CRD risk in the univariate analysis, no such association was observed when other risk factors were accounted for in the multivariate analysis (Table 1). More conclusively, the use of canned gas was not found to have any association in either univariate or multivariate analysis (Table 1).

In addition, while studies have shown that poverty and deprivation are associated with increased CRD risk, no such association was observed here (Table 1) (Axelsson Fisk & Merlo, 2017; Lee et al., 2019). While income often appears to be a dominant, sometimes even singular risk factor for illnesses in general, that is because it is a broadly relevant but nevertheless indirect and distal cause of most diseases, that operates indirectly through a diversity of more direct, proximal causes. It is therefore interesting that in this analysis, which accounts for variations in socioeconomic status at neighborhood but not household level, and also for fine‐scale variations in environmental risk factors, no association of CRD with this broadly important determinant of health was identified. While this might initially seem at odds with the existing evidence base, it may also be explicable in terms of the close association of poverty and CRD with environmental risk factors at scales of individual households and their immediate surroundings.

On the other hand, this well‐supported evidence, derived from geovalidation using high resolution satellite imagery and on‐the‐ground observations (ground truthing), not only validates the association between CRDs and hydrology, but also underscores the importance of not solely relying on coarse resolution DEMs data for studying hydrology, as fine scale features can be overlooked as seen in Figure 6. Hence in absence of fine resolution data, comprehensive validation methods are required to reach conclusion in this type of studies, considering the current observed evidence that shows fine scale hydrological processes may capture effect of CRD than TWI.

While place of residence is in principle a matter of personal choice, in practice, deprived households are often compelled to live clustered alongside each other in areas characterized by challenging hydrological conditions, due to socioeconomic factors and family history. For instance, deprived communities are typically composed largely of households who cannot afford housing in less vulnerable areas, forcing them to settle in known flood‐prone areas. Moreover, urbanization and population growth often lead to increased demand for housing, driving the expansion of settlements into floodplain areas, due to limited application of planning and development controls. More detailed fine‐scale data on relevant characteristics of locations and buildings, such as maximum flood level, moisture content, mold formation and airborne concentrations of fungal spores could therefore be important to record in future observational and interventional studies of the relationship between hydrology and CRD.

This study had several limitations that should be considered when interpreting the results. One significant limitation was the sample size, which may have affected the statistical power of the modeling. While the odds ratio confidence intervals for TWI and the number of household members were reasonably narrow, those for air conditioning and communal skip use were very wide, so it is certainly possible that some other important risk factors were missed. A larger sample size might therefore have provided more robust, insightful and reliable results. Additionally, the coarse resolution of socioeconomic status data, specifically related to poverty and deprivation at the neighborhood level, may well have obscured finer‐scale variations and nuances in these socioeconomic factors. While individual‐level data could potentially provide additional insight and statistical power, this study only collected household‐level data, which may limit the granularity of certain analyses but remains appropriate for assessing environmental influences on CRD.

## 5 Conclusion

Despite these study limitations, the results presented nevertheless indicate that hydrology is one of the most important drivers of CRD in the low‐lying, sandy, hot and humid environment of Dar es Salaam city. In addition to a number of known risk factors for CRD, this study identified a clear link between areas prone to accumulate and retain water, where houses and residential buildings are consequently likely to suffer from chronic dampness, mold proliferation and poor indoor air quality. The observed interaction between hydrological conditions and CRD further emphasizes the necessity for comprehensive urban planning and astute construction methods that address both environmental and public health concerns.

## Conflict of Interest

The authors declare no conflicts of interest relevant to this study.

## Figures and Tables

**Figure D1:**
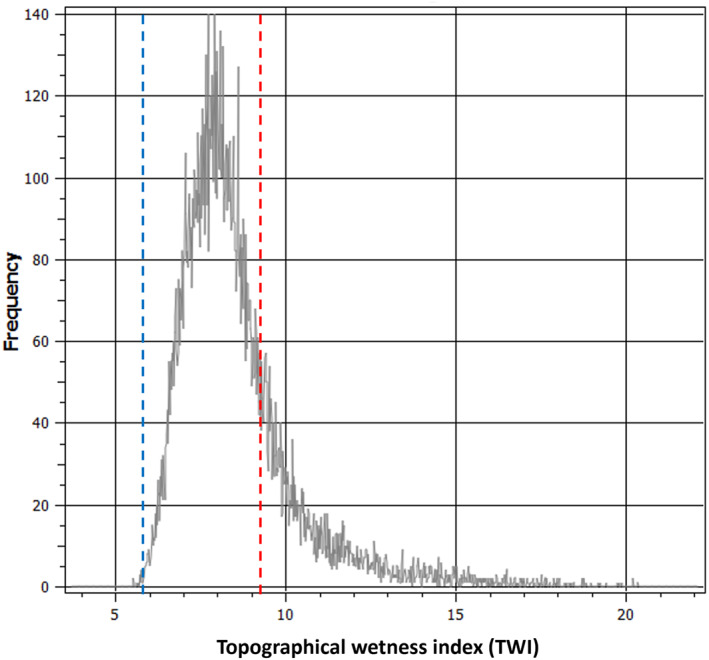
Raster histogram illustrating the frequency distribution of the topographical wetness index (TWI) within Dar es Salaam city, derived from a Digital Elevation Model (DEM) with a resolution of 30 m, a blue dotted line indicating tenth percentile (TWI ≈ 5.4), and a red dotted line indicating ninetieth percentile (TWI ≈ 9.2).

## Data Availability

The data supporting the findings of this study are openly available in GCRF Centre for Sustainable, Healthy and Learning Cities and Neighborhoods (Wang et al., 2023).

## Appendix A The Age and Sex Distribution of Residents in Dar es Salaam City Corresponds to That of the Sampled Households

**Table:**

	Males		Females		Total	
Age	*n*	%*	*n*	%*	*n*	%*
1–4	373	13.5	376	10.7	**749**	12.1
5–9	359	11.4	400	11	**759**	11.2
10–14	325	10.2	333	8.8	**658**	9.5
15–19	285	9.2	327	10	**612**	9.6
20–24	319	11.9	369	14	**688**	13
25–29	252	9.6	371	12	**623**	10.9
30–34	254	9.3	336	11	**590**	10.1
35–39	215	6.1	280	5.9	**495**	6
40–44	200	5.5	243	5.4	**443**	5.4
45–49	191	5	206	4.1	**397**	4.5
50–54	168	2.9	143	2	**311**	2.4
55–59	90	1.4	99	1.3	**189**	1.4
60–64	96	1.5	112	1.4	**208**	1.5
65–69	71	1.1	81	1	**152**	1
70–74	63	0.8	42	0.4	**105**	0.6
75–79	23	0.3	26	0.3	**49**	0.3
80+	18	0.3	52	0.6	**70**	0.5
Total	3,302	100	3,796	100	**7,098**	100

## Appendix B Number of Outcomes Events of Each Variable Investigated in the Current Study Stratified Into Income Categories Used in Sampling the Study Neighborhoods (Mitaa, Literally Translated as Streets)

**Table:**

Variables	Low	Mixed low and medium	Medium	Mixed medium and high	High	Total
Topographical wetness Index						
≤8	140	179	196	89	96	700
≥9	55	67	70	52	55	299
Chronic respiratory diseases	8	17	17	19	4	65
Number of People in the Household						
1–2	17	27	28	15	56	143
3–4	71	77	89	92	138	467
5+	170	207	209	219	118	923
Recent Birth in the Household	89	102	115	79	43	428
Any Air Conditioning	62	10	61	117	231	481
Use of Communal Skip Bins	2	1	12	5	1	21
Years Lived in Dwelling						
≤10	160	139	163	165	178	805
≥10	98	172	136	161	134	701
Cooking Energy						
Canned gas	83	83	154	263	279	862
Charcoal	164	204	139	51	18	576
Number of respondents	271	298	326	326	312	1,533

## Appendix C Demographic, Spatial and Socioeconomic Characteristics of Sampled Neighborhoods

**Table:**

S/n	District	Ward	Street	Population (Census 2,012)	Area (Kilometer square)	Population density people/Kilometer square	Income Category	No. household surveyed
1	Ilala	Kinyerezi	Kinyerezi	27,200	11.82	2300.48	Medium	135
2	Ilala	G/Mboto	Ulongoni	27,624	6.05	4562.41	Mixed low and medium	104
3	Kigamboni	Mjimwema	Mjimwema	7,413	4.17	1779.12	Medium	91
4	Kigamboni	Mjimwema	Kibugumo	4,217	8.91	473.45	Mixed low and medium	103
5	Kinondoni	Kawe	Mbezi Beach 'B'	16,531	4.40	3755.40	High	105
6	Kinondoni	Magomeni	Makuti 'A'	6,826	0.25	27596.75	Medium	100
7	Kinondoni	Kunduchi	Ununio	7,187	4.35	1650.46	Mixed medium and high	111
8	Kinondoni	Mikocheni	Regent Estate	2,989	1.08	2775.82	High	73
9	Kinondoni	Msasani	Masaki	7,619	3.98	1913.10	High	134
10	Temeke	Azimio	Mji Mpya	9,071	0.35	26153.12	Mixed low and medium	91
11	Temeke	Azimio	Kichangani	8,180	0.20	40350.63	Low	56
12	Ubungo	Kimara	Kimara Baruti	13,269	3.02	4393.78	Mixed medium and high	110
13	Ubungo	Manzese	Mwembeni	9,166	0.25	37016.40	Low	99
14	Ubungo	Goba	Goba	9,471	10.20	928.37	Mixed medium and high	105
15	Ubungo	Mburahati	Barafu	15,912	0.51	31263.02	Low	116

## Appendix D

Figure D1
